# Association and Interaction Analysis of Lipid Accumulation Product with Impaired Fasting Glucose Risk: A Cross-Sectional Survey

**DOI:** 10.1155/2019/9014698

**Published:** 2019-10-28

**Authors:** Jian Song, Xue Chen, Yuhong Jiang, Jing Mi, Yuyuan Zhang, Yingying Zhao, Xuesen Wu, Huaiquan Gao

**Affiliations:** ^1^School of Public Health, Bengbu Medical College, 2600 Donghai Road, Bengbu, 233000 Anhui Province, China; ^2^Bengbu Health Board, 568 Nanhu Road, Bengbu, 233000 Anhui Province, China

## Abstract

**Aims:**

Lipid accumulation product (LAP) is put forward as a powerful marker showing the accumulation of visceral fat. The present study is aimed at (i) analyzing the predictive performances of LAP in the identification of impaired fasting glucose (IFG) in Chinese population and (ii) exploring the potentially interactive effect between LAP and other factors on IFG risk.

**Methods:**

Analysis was conducted on the data obtained from a community-based cross-sectional survey in Chinese population, and all the participants enrolled were required to complete a face-to-face questionnaire survey and related health checks. Then, for the purpose of comparing predictive values between LAP and conventional obesity indices for IFG, relevant analysis was carried out on the receiver operating characteristic (ROC) curve. The assessment of interactive effects was conducted by employing the three indicators as follows: (1) RERI (the relative excess risk due to interaction), (2) AP (attributable proportion due to interaction), and (3) SI (synergy index).

**Results:**

A total of 1777 participants (748 males and 1029 females) were involved in the final analysis. It was finally obtained that the prevalence rate of IFG was 14.1% in total, 15.5% for males and 13.1% for females, respectively. In logistic regression analysis, individuals with LAP levels in the fourth quartile had a significant higher risk of getting IFG in comparison with that of the lowest quartile (crude OR: 4.58, 95% CI: 3.01-6.98; adjusted OR: 3.81, 95% CI: 2.33-6.23). In addition, it was indicated by the ROC curve analysis that LAP showed a better performance in discriminating IFG risk than BMI in both males (*Z* = 2.20, *P* = 0.028) and females (*Z* = 2.13, *P* = 0.033). However, LAP displayed a higher predictability in comparison with WC only in females (*Z* = 2.07, *P* = 0.038), but not in males (*Z* = 0.18, *P* = 0.860). Furthermore, LAP and family history of diabetes were able to impose significant synergistic interaction on the risk of IFG, which was indicated by all the parameters in females (RERI: 2.52, 95% CI: 0.19-4.84; AP: 0.47, 95% CI: 0.20-0.74; SI: 2.39, 95% CI: 1.17-4.87) and males (RERI: 2.18, 95% CI: 0.08-4.73; AP: 0.43, 95% CI: 0.07-0.79; SI: 2.15, 95% CI: 1.03-5.45). However, none of the indicators showed significant interaction between LAP and smoking on the risk of IFG in females (RERI: 0.92, 95% CI: -2.79-4.63; AP: 0.20, 95% CI: -0.50-0.92; SI: 1.37, 95% CI: 0.42-4.52). Meanwhile, there was also no significant interaction between LAP and smoking on the risk of IFG in males as indicated by the value of SI (2.22, 95% CI: 0.80-6.21).

**Conclusion:**

It was concluded that LAP was significantly related to a higher risk of IFG in Chinese population, and its performance was superior to that of conventional obesity indices, especially in females. Apart from that, LAP with family history of diabetes may have an interactive effect that can impose a great influence on the development of IFG.

## 1. Introduction

It is widely acknowledge that impaired fasting glucose (IFG), as a state of prediabetes, is a manifestation of glucose metabolism disorders in the early period [1]. It was currently reported that China has a population of nearly 148.2 million adults suffering from prediabetes [2]. Moreover, it is more likely for individuals with IFG to further progress to diabetes [3]. It has been shown by a large amount of evidence that IFG might lead to the significantly increased incidence of a series of cardiovascular events [4–6]. In the meantime, IFG was closely related to the increased clustering of cardiovascular risk factors [7]. Besides, it was reported that the cumulative incidence of hypertension was substantially higher in individuals with IFG as compared with normal fasting glucose collected from the cohort study on Chinese in rural areas [8]. A 10-year prospective study carried out in Eastern China indicated that IFG significantly increased the risk of all-cause mortality among Chinese adults [9]. As a form of diabetes in the period of predevelopment, the role played by IFG has been attracting increasing attention in the fields of disease prevention and mortality reduction. Therefore, it is of vital importance to have a comprehensive understanding of the risk factors possessed by IFG and their possibly interactive effect.

Obesity is known as a serious problem in the field of public health as considerable studies have demonstrated that it is closely linked to a higher risk of multiple chronic diseases, including glucose metabolism disorders [10]. Accelerated by the rapid development of society and economy, the increase of obesity is dramatically prevalent especially in developing countries [11]. It was emphasized that the effect of obesity on glucose was related to not only fat content but also the distribution of the excess adiposity [12, 13]. As far as structure and function are concerned, obvious differences are available in subcutaneous adipose tissue (SAT) and visceral adipose tissue (VAT), and it seems that visceral obesity is able to impose more substantial effect on unfavorable outcomes [14, 15]. However, it is unable for conventional obesity indices, such as body mass index (BMI) and waist circumference (WC), to reflect accumulation of visceral fat accurately [14]. In the meanwhile, the gold standards used to measure visceral fat by employing the methods of imaging are not readily available in daily clinical practice and epidemiological survey because of financial burden and radiation exposure. Therefore, an available and efficient marker which can reflect visceral obesity is able to impose great importance. It was suggested that lipid accumulation product (LAP) based on WC and triglycerides (TG) can be used to estimate the accumulation of visceral fat [16]. It was also identified that increased LAP was significantly correlated with a higher level of serum total cholesterol, fasting plasma glucose, and insulin, and it was negatively related to high-density cholesterol (HDL) [17]. In addition, a significant relationship was observed between LAP and insulin resistance [18]. Moreover, when discriminating the risk of metabolic syndrome, LAP was shown to perform better in comparison with BMI and WC [19]. Besides, it was indicated by a population-based cohort survey that LAP displayed a better predictive ability in the 10-year cardiovascular disease incidence as compared with common obesity indices [20]. However, no prior work has been done to explore the performance of LAP in the identification of IFG, as well as its comparison with other obesity indicators.

Additionally, as IFG is a heterogeneous metabolic disorder resulting from a combination of genetic and environmental factors, the joint influence imposed by risk factors may aggravate the risk of IFG. The National Health and Nutrition Examination Survey (NHANES) reported that there was a significant interactive effect of abdominal obesity and 25-hydroxyvitamin D on the risk of insulin resistance [21]. It was demonstrated by a prospective cohort study with the participants of 3598 Chinese adults that smoking and abdominal obesity interacted with synergistically influenced the risk related to diabetes of type 2 [22]. Furthermore, a significant interaction was available between the LAP with family history of hypertension and the risk of hypertension [23]. However, few studies have analyzed the interactive effect between risk factors on IFG.

In summary, this study is aimed (1) at analyzing the association between LAP and risk of IFG in Chinese population, (2) at comparing the performance of LAP with the indicators of conventional obesity in the prediction of IFG, and (3) at exploring the possible interactive effect between LAP and other risk factors on IFG.

## 2. Methods

### 2.1. Study Design

The data employed in this study was collected from the “Creation of Provincial-level Chronic Diseases Management Demonstration Zone” project launched in Longzihu, Bengbu, Anhui Province, China, from July to August 2015. The project was supported by Bengbu Health Board and approved by the Ethics Committee of Bengbu Medical College. Participants of this project were selected randomly by employing a method of multistage sampling. In brief, this survey was conducted in seven residential communities of Longzihu, Bengbu, and individuals who were diagnosed with neuropathy previously or had no ability to finish this survey independently were excluded by us in this survey. For the selected individuals, they were firstly required to complete relevant questionnaires face to face and then both anthropometric tests and laboratory examinations were carried out for each of them. All the survey projects were completed in Community Health Center, and informed consent was signed by all the participants of this study.

### 2.2. Survey and Measurements

The questionnaire survey designed by us was conducted for each of the participants by trained interviewers. Sociocharacteristics mainly included the factors as follows: age, gender, marital status, educational level, smoking, and history of diseases. Among the information investigated, smoking was defined as the status of presmoking or current smoking in detail. Moreover, individuals who had at least one parent or sibling with diabetes of type 2 were deemed as having the family history of diabetes. In terms of marital status, it was composed of currently married and not married. As for educational level, it was further categorized into two groups: “middle school graduate or lower” and “high school graduate or university.” As far as income was concerned, it was also classified into two groups: “less than 4000” and “greater or equal to 4000.”

It was required by the height measurement that the subject should remove the shoes, hat, jacket, and wear light clothing. During the process of measurement, the subject was required to come close to the measuring scale with two heels close to each other and the measurement had the error of 0.1 cm. When measuring WC, the subject should be fasting, upright the body, and relax the abdomen with a distance of 30~40 cm between the two feet. A soft ruler was placed in the midpoint of the line connecting the upper edge of the tibia at the midaxillary line of the right side and the lower edge of the twelfth rib, and then it should circle the abdomen in the horizontal direction. Finally, fasting plasma glucose (FPG) and triglycerides (TG) were examined by collecting blood samples from each of the participants after an overnight fast (≥ eight hours).

### 2.3. Relevant Definitions

1. Diabetes: defined as a fasting plasma glucose (FPG) value ≥7.0 mmol/L or a previous diagnosis of type 2 diabetes [24]
2. IFG: according to the 1999 the World Health Organization (WHO) diagnostic criteria [25] and the Guideline for the Prevention and Treatment of Diabetes Mellitus in China (2017) [24], IFG was defined as a fasting plasma glucose value of 6.1 mmol/L ≤ FPG < 7.0 mmol/L
3. Normal fasting glucose (NFG): defined as FPG < 6.1 mmol/L and without history of diagnosed diabetes [24]
4. General obesity: BMI (weight(kg)/height(m)^2^) ≥ 28 was regarded as general obesity [26]
5. Abdominal obesity: WC ≥ 90 cm for males and ≥85 cm for females [27]
6. LAP: calculated as [WC(cm) − 65] × [TG(mmol/L)] for males and [WC (cm) − 58] × [TG(mmol/L)] for females [16]

### 2.4. Statistical Methods

Description of the categorical variables was conducted by percentages, and comparison was conducted by the chi-squared test or Kruskal-Wallis *H* test between three glycaemia groups (NFG, IFG, and diabetes). In terms of LAP level, it was classified into four groups (Q1, Q2, Q3, and Q4) by quartiles. Comparison of the age between groups was conducted by the Kruskal-Wallis *H* test. During the analysis on the risk factors of IFG, logistic regression was carried out by calculating odds ratio (OR) and its corresponding 95% confidence interval (95% CI). For the purpose of obtaining predictive values between LAP and conventional obesity indices for IFG, receiver operating characteristic (ROC) curve analysis was conducted by us through the calculation of the area under ROC curve (AUC). Finally, the following indicators were employed for the aim of assessing the interaction between LAP and other factors on IFG risk: (1) the relative excess risk due to interaction (RERI): it was significant if the 95% CI did not overlap 0; (2) the proportion of interaction due to interaction (AP): it was significant if the 95% CI did not overlap 0; and (3) synergy index (SI): it was significant if the 95% CI did not overlap 1 [28, 29]. The following factors were adjusted in the logistic regression as well as in the interaction analysis: age, gender, educational level, income, marital status, smoking, and family history of diabetes and obesity. R software was applied for the completion of all the statistical analysis, and the threshold for statistical significance was set as *α* = 0.05.

## 3. Results

### 3.1. General Characteristics of Study Population

The general characteristics of the study population were specified in detail in Table 1. A total of 1777 participants (748 males and 1029 females) were enrolled in the final analysis. On the whole, the prevalence rate of IFG was 14.1%. There were 116 (15.5%) IFG members in male and 135 (13.1%) IFG members in female. In addition, the mean ages obtained from the NFG, IFG, and diabetes groups were 59.5 ± 11.26, 1.9 ± 11.7, and 65.0 ± 10.0, respectively. Moreover, significant differences of age were available between the groups (*H* = 32.5, *P* < 0.001). Meanwhile, in comparison with the NFG, IFG, and diabetes groups, significant differences were observed in the following indicators of educational level (*χ*^2^ = 7.9, *P* = 0.020), smoking rate (*χ*^2^ = 28.3, *P* < 0.001), family history of diabetes (*χ*^2^ = 25.1, *P* < 0.001), general obesity (*χ*^2^ = 45.0, *P* < 0.001), abdominal obesity (*χ*^2^ = 64.0, *P* < 0.001), and LAP quartiles (*χ*^2^ = 131.8, *P* < 0.001). However, there were no statically significant differences in terms of marital status (*χ*^2^ = 0.5, *P* = 0.795) and income (*χ*^2^ = 0.4, *P* = 0.836).

### 3.2. Risk Factors for IFG

Table 2 shows the results obtained from logistic regression analysis. In univariate analysis, male had a higher risk of getting IFG (OR: 1.33, 95% CI: 1.01-1.74) in comparison with female. However, this significant influence disappeared (OR: 0.88, 95% CI: 0.61-1.26) after the implementation of adjustment for other factors. In addition, the risk of IFG increased significantly with the increase of age in multivariate analysis (OR: 1.02, 95% CI: 1.01-1.03). Besides, individuals with smoking behavior had higher risks of suffering from IFG compared with nonsmokers (crude OR: 2.00, 95% CI: 1.51-2.65; adjusted OR: 1.98, 95% CI: 1.38-2.83). Apart from that, family history of diabetes also showed significant association with the risk of IFG (crude OR: 1.62, 95% CI: 1.15-2.27; adjusted OR: 1.55, 95% CI: 1.07-2.24). In two logistic models, the factors of educational level (crude OR: 0.75, 95% CI: 0.55-1.02; adjusted OR: 0.80, 95% CI: 0.57-1.11), marital status (crude OR: 0.91, 95% CI: 0.63-1.30; adjusted OR: 1.07, 95% CI: 0.71- 1.61), and income (crude OR: 1.14, 95% CI: 0.62-2.13; adjusted OR: 1.17, 95% CI: 1.60-2.30) were all not significantly related to the risk of IFG. As far as obesity indicators are concerned, general obesity (crude OR: 2.33, 95% CI: 1.68-3.25; adjusted OR: 0.60, 95% CI: 1.11-2.32) could stimulate the prevalence of IFG substantially. However, abdominal obesity showed significant association with IFG in univariate analysis (crude OR: 2.08, 95% CI: 1.58-2.74), but not in multivariate analysis (adjusted OR: 1.02, 95% CI: 0.72-1.44). As for LAP, the risk of IFG increased significantly with the increase of its levels in the fourth quartile as compared with the bottom quartile (crude OR: 4.58, 95% CI: 3.01-6.98; adjusted OR: 3.81, 95% CI: 2.33-6.23).

### 3.3. ROC Curve Analysis

We made comparison between the predictive abilities of various obesity indices in IFG risk, and the results are shown in Table 3 accordingly. Overall, the AUCs (95% CI) of BMI, WC, and LAP were 0.61 (0.59-0.64), 0.63 (0.61-0.66), and 0.68 (0.65-0.70), respectively. LAP was superior to BMI (*Z* = 3.26, *P* = 0.001) and WC (*Z* = 2.40, *P* = 0.016) in predicting IFG risk. As for females, it was shown that the AUCs (95% CI) of BMI, WC, and LAP were 0.59 (0.56-0.62), 0.61 (0.58-0.65), and 0.66 (0.63-0.69), respectively. LAP also showed a better performance in discriminating IFG compared with BMI (*Z* = 2.13, *P* = 0.033) and WC (*Z* = 2.07, *P* = 0.038). As for males, LAP had a higher predictability as compared with BMI (*Z* = 2.20, *P* = 0.028), with AUC (95% CI) of 0.66 (0.61-0.69) and 0.59 (0.55-0.63), respectively. However, there was no significant difference (*Z* = 0.18, *P* = 0.860) shown between the AUC of LAP and WC (0.65, 95% CI: 0.61-0.69).

### 3.4. Analysis on Interaction

Table 4 shows the results obtained from the analysis on interaction. Firstly, the individuals were classified into four groups based on the status of LAP and family history of diabetes. In females, a combination of high LAP and positive family history of diabetes showed a significantly higher risk of IFG in comparison with members with low LAP and nonfamily history of diabetes (OR: 5.33, 95% CI: 3.32-8.55). Besides, after the implementation of adjustment conducted for other factors, three measurements of additive interaction were all significant: RERI: 2.52, 95% CI: 0.19-4.84; AP: 0.47, 95% CI: 0.20-0.74; SI: 2.39, 95% CI: 1.17-4.87. Similarly, in males, the value RERI (2.18, 95% CI: 0.08-4.73), AP (0.43, 95% CI: 0.07-0.79), and SI (2.15, 95% CI: 1.03-5.45) all indicated a significant synergistic interaction between LAP and family history of diabetes on the risk of IFG. Then, the interaction between LAP and smoking on the risk of IFG was further analyzed, and it was indicated by the results that the LAP (+) and smoking (+) group had the highest prevalence of IFG in comparison with the reference group both in females (OR: 4.38, 95% CI: 1.93-9.96) and males (OR: 4.62, 95% CI: 2.19-9.73). While, none of the three indicators showed significant interaction between LAP and smoking on IFG risk in females (RERI: 0.92, 95% CI: -2.79-4.63; AP: 0.20, 95% CI: -0.50-0.92; SI: 1.37, 95% CI: 0.42-4.52). As for males, only the values of RERI (1.99, 95% CI: 0.05-3.94) and AP (0.43, 95% CI: 0.10-0.77), rather than the value of SI (2.22, 95% CI: 0.80-6.21), were statistically significant. Therefore, there was also no significant interaction between LAP and smoking on the risk of IFG in males.

## 4. Discussion

In this population-based survey, it was demonstrated that IFG, with a rate of 14.1%, was prevalent in Chinese population. In addition, the prevalence of IFG varied across the studies as two different definitions of IFG, WHO criteria and the criteria established by American Diabetes Association (ADA, 5.6-6.9 mmol/L), were available [30]. Because diagnostic criteria established by WHO for IFG are adopted currently in daily practice in China, this study employed the diagnostic criteria of WHO to define IFG [24]. In comparison with other studies which used the criteria of WHO in China, a survey conducted in Jiangsu Province with 19705 participants showed the same prevalence (14.1%) of IFG with our study [31]. However, participants living in Jilin and Xinjiang Province showed a relatively lower rate of IFG (11.8% and 10.0%, respectively) [32, 33]. Besides, a large-scale epidemiological survey carried out in Liaoning Province reported that the prevalence of IFG was 16.5% [34]. As China is a vast country which covers a total area of over 9.6 million square kilometers, the levels of economic development and differences existing in culture and lifestyles may influence the epidemic of IFG.

It was suggested by the present study that LAP showed a significant correlation with a higher risk of IFG. Similarly, Wang et al. [35] conducted a survey on the population of 11113 Chinese in rural area with a follow-up of six years, and it was indicated by the results that the fourth quartile of LAP had 5-fold risks in male and 6-fold risks in female of getting diabetes in comparison with the first quartile of LAP, respectively. Besides, it was demonstrated by a cohort study that LAP showed significant association with incident diabetes of type 2 in the population of Korea [36]. Furthermore, LAP showed a higher predictability in comparison with BMI in terms of IFG risk in both genders. BMI was known as a frequently used indicator for relative weight, but it was unable to distinguish between adipose and lean tissue. In multivariable Cox proportional model, LAP instead of BMI served as an independent predictor of all-cause mortality in individuals with high cardiovascular risk [37]. The ROC curve analysis also indicated that LAP performed better than BMI in predicting metabolic syndrome and prehypertension among Chinese individuals [38, 39]. However, LAP only showed a better performance than WC in female when discriminating IFG risk, suggesting that the relation between LAP and IFG varies when different genders are concerned. The gender differences may be caused by the accumulation and metabolism of free fatty acids which are different according to different genders, and these lipids made more contributions to increased VAT volume in women instead of men [39, 40]. Another potential explanation for the gender difference lies in the fact that the patterns of lipid over accumulation became more and more distinct with the aging of different genders [41]. At older ages, the changes of annual LAP became greater with the aging of women, while the changes decreased gradually for male [41]. Similarly, LAP was only superior to WC in females in discriminating the risk of prehypertension in Chinese population [42]. It was found that LAP was independently related to diabetes of type 2 in women, but not men, with hypertension [43]. Compared with obese men, a stronger relationship was available between VAT with more than one cardiometabolic risk factor in obese women [44]. It was reported by a cross-sectional study with a total of 54477 Japanese participants that the ORs were significant for risk of both the diabetes and hyperglycemia, but it seemed to be higher in female than male [45]. In addition, a statistically significant relationship was available between the increased level of LAP and a higher risk of intracranial atherosclerotic stenosis in Chinese female ≥40 years old, but this relationship in Chinese male was not significant [46]. LAP seemed to be more valuable in females, but the reasons of gender-specific differences are not fully clear.

Excess VAT and SAT serve as two important manifestations of abdominal obesity, but obvious difference between them is available in their structural composition and molecular biology [14, 15]. As for why VAT was more closely related to diseases risk, it can be explained by potential mechanisms. Firstly, the lipolytic activity is stronger in VAT compared with that of SAT [47]. Secondly, the cytokines released by the two fat depots are also differential and SAT is less involved in obesity-related metabolic disorders [14, 15]. The Framingham Heart Study demonstrated that VAT had a stronger relationship with insulin resistance in comparison with SAT [48]. In Chinese subjects with prediabetes, there was also a close relationship between VAT, instead of SAT, and insulin resistance [49]. Compared with total body fat and SAT, VAT was most strongly associated with the deficiency of vitamin D [50]. When comparing the abilities of various measures of body fat in identifying the risk of chronic kidney diseases, it was shown that only VAT was a significantly independent risk factor in multivariable analysis [51]. In addition, a stronger relationship was available between VAT, rather than SAT, and cardiometabolic risk factors [52]. Moreover, Chinese individuals have a greater amount of VAT than Europeans at a same level of BMI or WC [53]. Therefore, an easily obtainable and accurate measurement of visceral fat accumulation is demanding especially in Chinese population.

LAP, which is composed of one anthropometric and one biochemical parameter, has a theoretical basis in reflecting the accumulation of visceral fat [16]. WC is a parameter that can reflect abdominal adipose tissue, but it cannot determine the degree of visceral fat accumulation in the body. It was discovered that a significant relationship was available between a higher WC and insulin resistance, as well as IFG and metabolic syndrome [54, 55]. It was demonstrated by cohort studies that WC was an independent factor that is associated with the risk of cardiovascular diseases [56–58]. On the other hand, a significant relation was available between TG concentrations and insulin resistance [59] and the increased TG was independently associated with the risk of hypertension, diabetes, and IFG [60, 61]. Moreover, TG was demonstrated to be significantly associated with VAT in healthy men, even after controlling for SAT [62]. Thus, it is appealing to take into consideration both WC and TG that can reflect the accumulation of visceral fat. “HyperTG-waist (HTGW),” as a dichotomous indicator, is a combination of WC and TG. The rural Chinese cohort study of 12086 individuals with a median follow-up of 6 years demonstrated that HTGW was a major risk factor existing in type 2 diabetes [63]. In a cross-sectional survey conducted for a total of 12757 adults aged 40 years and older, it was proved that HTGW phenotype was a remarkable risk factor and practical screening tool of prediabetes [64]. However, a continuous marker for the accumulation of visceral fat may be more accurate and valuable as obesity itself is a continuous process [14]. It was also observed that there was a strong correlation between LAP and the area of VAT measured by CT in a cross-sectional survey [65].

It was shown by interaction analysis that there was a significant interaction between LAP and family history of diabetes on the risk of IFG. Family history of diabetes is considered to be an easily identifiable marker of genetic susceptibility and its significant correlation with the risk of diabetes and prediabetes was reported [66, 67]. Similarly, Sargeant et al. [68] conducted relevant investigation on the significant influence imposed by family history of diabetes and obesity on diabetes in middle-aged and elderly individuals. It was shown by a cross-sectional study in Sweden that family history of diabetes and obesity had an interactive influence on prediabetes, instead of diabetes, in females [69]. However, no significant interaction between LAP and smoking was observed in both genders. Previous studies have also explored the interactive effect between smoking and obesity on risk of diabetes, but the results seem to be inconsistent. For instance, a population-based prospective cohort of 3598 Chinese individuals demonstrated that there was a significant interaction of current smoking and abdominal obesity on type 2 diabetes risk [22]. But another community-based cohort study reported that there was no combined influence of BMI and smoking on the risk of diabetes in females [70].

Limitations of this paper should be taken into account. Firstly, it is unable to for cross-sectional survey to infer causality in its results. Secondly, this study was conducted in Chinese population, but there are ethnic differences in body composition as mentioned above, and therefore, whether LAP has better performance in identifying IFG in other race is not clear. Thirdly, it was demonstrated that the influence of LAP on human beings may be subjected to the factor of age [71], but the enrolled individuals in this survey were all middle-aged and elderly.

## 5. Conclusion

These results showed that LAP serves as a simple and powerful tool that can be employed in clinical practice and epidemiology survey. In addition, increased LAP was significantly related to a higher risk of IFG in Chinese population, and it had a better performance than conventional obesity indices especially in females. Moreover, LAP with family history of diabetes may have an interactive effect that can influence the development of IFG. Therefore, it is quite necessary for future studies to focus on the underlying mechanisms of interactive effect on IFG risk.

## Figures and Tables

**Table 1:** Basic characteristic of study participants.

Variables	NFG (*N* = 1211)	IFG (*N* = 251)	Diabetes (*N* = 315)	*H*/*χ*^2^	*P* ^a^
Age (years)	59.5 ± 11.2	61.9 ± 11.7	65.0 ± 10.0	32.5	<0.001^1^
Gender (male%)	476 (39.3)	116 (46.2)	156 (49.5)	12.7	0.002^2^
Educational level (*n*(%))				7.9	0.020^2^
Middle school graduate or lower	815 (67.3)	184 (73.3)	234 (74.3)		
High school graduate or university	396 (32.7)	67 (26.7)	81 (25.7)		
Marital status (*n*(%))				0.5	0.795^2^
Currently married	1025 (84.6)	209 (83.3)	263 (83.5)		
Currently not married	186 (15.4)	42 (16.7)	52 (16.5)		
Income (yuan) (*n*(%))				0.4	0.836^2^
<4000	1156 (95.5)	238 (94.8)	302 (95.9)		
≥4000	55 (4.5)	13 (5.2)	13 (4.1)		
Smoking (*n*(%))	313 (25.8)	103 (41.0)	110 (34.9)	28.3	<0.001^2^
Family history of diabetes (*n*(%))	179 (14.8)	55 (21.9)	82 (26.0)	25.1	<0.001^2^
General obesity (*n*(%))	152 (12.6)	63 (25.1)	80 (25.4)	45.0	<0.001^2^
Abdominal obesity (*n*(%))	480 (39.6)	145 (57.8)	189 (60.0)	64.0	<0.001^2^
LAP (*n*(%))				131.8	<0.001^1^
Q1	365 (30.1)	39 (15.5)	40 (12.7)		
Q2	338 (27.9)	46 (18.3)	62 (19.7)		
Q3	292 (24.1)	66 (26.3)	85 (27.0)		
Q4	216 (17.8)	100 (29.8)	128 (40.6)		

^1^Kruskal-Wallis *H* test. ^2^Chi-squared test.

**Table 2:** Logistic regression model for risk factors associated with IFG.

Variables	Crude		Adjusted	
	OR (95% CI)	*P*	OR (95% CI)	*P*
Gender				
Female	1 (ref)	—	1 (ref)	—
Male	1.33 (1.01-1.74)	0.043	0.88 (0.61-1.26)	0.472
Age (years)	1.02 (1.01-1.03)	0.002	1.02 (1.01-1.03)	0.004
Educational level				
Middle school graduate or lower	1 (ref)	—	1 (ref)	—
High school graduate or university	0.75 (0.55-1.02)	0.063	0.80 (0.57-1.11)	0.178
Marital status				
Currently married	1 (ref)	—	1 (ref)	—
Currently not married	0.91 (0.63-1.30)	0.585	1.07 (0.71-1.61)	0.764
Income (yuan)				
<4000	1 (ref)	—	1 (ref)	—
≥4000	1.14 (0.62-2.13)	0.671	1.17 (0.60-2.30)	0.643
Smoking				
No	1 (ref)	—	1 (ref)	—
Yes	2.00 (1.51-2.65)	<0.001	1.98 (1.38-2.83)	<0.001
Family history of diabetes				
No	1 (ref)	—	1 (ref)	—
Yes	1.62 (1.15-2.27)	0.005	1.55 (1.07-2.24)	0.021
General obesity				
No	1 (ref)	—	1 (ref)	—
Yes	2.33 (1.68-3.25)	<0.001	1.60 (1.11-2.32)	0.012
Abdominal obesity				
No	1 (ref)	—	1 (ref)	—
Yes	2.08 (1.58-2.74)	<0.001	1.02 (0.72-1.44)	0.910
LAP				
Q1	1 (ref)	—	1 (ref)	—
Q2	1.35 (0.84-2.18)	0.222	1.32 (0.81-2.17)	0.380
Q3	2.22 (1.29-3.17)	0.002	1.96 (1.20-3.19)	0.007
Q4	4.58 (3.01-6.98)	<0.001	3.81 (2.33-6.23)	<0.001

**Table 3:** The comparisons of various obesity indices in predicting IFG risk.

Obesity indicators	All			Male			Female		
	AUC (95% CI)	*Z*	*P* ^1^	AUC (95% CI)	*Z*	*P* ^1^	AUC (95% CI)	*Z*	*P* ^1^
BMI	0.61 (0.59-0.64)	3.26	0.001	0.59 (0.55-0.63)	2.20	0.028	0.59 (0.56-0.62)	2.13	0.033
WC	0.63 (0.61-0.66)	2.40	0.016	0.65 (0.61-0.69)	0.18	0.860	0.61 (0.58-0.65)	2.07	0.038
LAP	0.68 (0.65-0.70)	—	—	0.66 (0.61-0.69)	—	—	0.66 (0.63-0.69)	—	—

^1^Compared with LAP.

**Table 4:** Index of interactive effect between LAP and other factors on IFG.

Categories		Female		Male	
		OR (95% CI)^2^	Index of interactive effect^2^	OR (95% CI)^2^	Index of interactive effect^2^
LAP^1^	Family history of diabetes				
-	-	1 (ref)		1 (ref)	
-	+	1.17 (0.63-2.17)	RERI: 2.52 (0.19-4.84)^3^	1.61 (0.71-3.67)	RERI: 2.18 (0.08-4.73)^3^
+	-	2.65 (1.92-3.65)	AP: 0.47 (0.20-0.74)^3^	2.28 (1.28-4.07)	AP: 0.43 (0.07-0.79)^3^
+	+	5.33 (3.32-8.55)	SI: 2.39 (1.17-4.87)^3^	5.07 (2.62-8.39)	SI: 2.15 (1.03-5.45)^3^
LAP^1^	Smoking				
-	-	1 (ref)		1 (ref)	
-	+	1.49 (0.65-3.42)	RERI: 0.92 (-2.79-4.63)^4^	1.59 (0.69-3.66)	RERI: 1.99 (0.05-3.94)^3^
+	-	2.97 (1.85-4.77)	AP: 0.20 (-0.50-0.92)^4^	2.03 (0.91-4.52)	AP: 0.43 (0.10-0.77)^3^
+	+	4.38 (1.93-9.96)	SI: 1.37 (0.42-4.52)^4^	4.62 (2.19-9.73)	SI: 2.22 (0.80-6.21)^4^

^1^Grouped by cut-off values calculating through ROC curve analysis. ^2^Multivariate analysis. ^3^*P* < 0.05. ^4^*P* > 0.05.

## Data Availability

The datasets used and/or analyzed during the current study are available from the corresponding authors on reasonable request.

## References

[1] Nathan D. M., Davidson M. B., DeFronzo R. A. (2007). Impaired fasting glucose and impaired glucose tolerance: implications for care. *Diabetes Care*.

[2] Xu Y., Wang L. M., He J. (2013). Prevalence and control of diabetes in Chinese adults. *Journal of the American Medical Association*.

[3] Admiraal W. M., Holleman F., Snijder M. B. (2014). Ethnic disparities in the association of impaired fasting glucose with the 10-year cumulative incidence of type 2 diabetes. *Diabetes Research and Clinical Practice*.

[4] Kim H. K., Kim C. H., Kim E. H. (2013). Impaired fasting glucose and risk of cardiovascular disease in Korean men and women: the Korean Heart Study. *Diabetes Care*.

[5] Levitzky Y. S., Pencina M. J., D'Agostino R. B. (2008). Impact of impaired fasting glucose on cardiovascular disease: the Framingham Heart Study. *Journal of the American College of Cardiology*.

[6] Levitan E. B., Song Y., Ford E. S., Liu S. (2004). Is nondiabetic hyperglycemia a risk factor for cardiovascular disease? A meta-analysis of prospective studies. *Archives of Internal Medicine*.

[7] Valentino G., Kramer V., Orellana L. (2015). Impaired fasting glucose in nondiabetic range: is it a marker of cardiovascular risk factor clustering?. *Disease Markers*.

[8] Zhao Y., Sun H., Wang B. (2017). Impaired fasting glucose predicts the development of hypertension over 6 years in female adults: results from the rural Chinese cohort study. *Journal of Diabetes and its Complications*.

[9] Shi Z., Zhen S., Zimmet P. Z. (2016). Association of impaired fasting glucose, diabetes and dietary patterns with mortality: a 10-year follow-up cohort in Eastern China. *Acta Diabetologica*.

[10] Verma S., Hussain M. E. (2017). Obesity and diabetes: an update. *Diabetes and Metabolic Syndrome: Clinical Research and Reviews*.

[11] Wu J., Xu H., He X. (2017). Six-year changes in the prevalence of obesity and obesity-related diseases in Northeastern China from 2007 to 2013. *Scientific Reports*.

[12] Fox C. S., Massaro J. M., Hoffmann U. (2007). Abdominal visceral and subcutaneous adipose tissue compartments: association with metabolic risk factors in the Framingham Heart Study. *Circulation*.

[13] Canoy D., Boekholdt S. M., Wareham N. (2007). Body fat distribution and risk of coronary heart disease in men and women in the European Prospective Investigation into Cancer and Nutrition in Norfolk cohort: a population-based prospective study. *Circulation*.

[14] Ibrahim M. M. (2010). Subcutaneous and visceral adipose tissue: structural and functional differences. *Obesity Reviews*.

[15] Abraham T. M., Pedley A., Massaro J. M., Hoffmann U., Fox C. S. (2015). Association between visceral and subcutaneous adipose depots and incident cardiovascular disease risk factors. *Circulation*.

[16] Kahn H. S. (2005). The “lipid accumulation product” performs better than the body mass index for recognizing cardiovascular risk: a population-based comparison. *BMC Cardiovascular Disorders*.

[17] Rotter I., Ryl A., Szylinska A., Pawlukowska W., Lubkowska A., Laszczyńska M. (2017). Lipid accumulation product (LAP) as an index of metabolic and hormonal disorders in aging men. *Experimental and Clinical Endocrinology & Diabetes*.

[18] Mazidi M., Kengne A. P., Katsiki N., Mikhailidis D. P., Banach M. (2018). Lipid accumulation product and triglycerides/glucose index are useful predictors of insulin resistance. *Journal of Diabetes and its Complications*.

[19] Ray L., Ravichandran K., Nanda S. K. (2018). Comparison of lipid accumulation product index with body mass index and waist circumference as a predictor of metabolic syndrome in Indian population. *Metabolic Syndrome and Related Disorders*.

[20] Kyrou I., Panagiotakos D. B., Kouli G. M. (2018). Lipid accumulation product in relation to 10-year cardiovascular disease incidence in Caucasian adults: the ATTICA study. *Atherosclerosis*.

[21] Kabadi S. M., Lee B. K., Liu L. (2012). Joint effects of obesity and vitamin D insufficiency on insulin resistance and type 2 diabetes: results from the NHANES 2001-2006. *Diabetes Care*.

[22] Luo W., Guo Z., Wu M., Hao C., Zhou Z., Yao X. (2015). Interaction of smoking and obesity on type 2 diabetes risk in a Chinese cohort. *Physiology & Behavior*.

[23] Song J., Zhao Y., Nie S., Chen X., Wu X., Mi J. (2018). The effect of lipid accumulation product and its interaction with other factors on hypertension risk in Chinese Han population: a cross-sectional study. *PLoS One*.

[24] Chinese Diabetes Society (2018). The guideline for the prevention and treatment of diabetes mellitus in China (2017). *Chinese Journal of Practical Internal Medicine*.

[25] Alberti K. G., Zimmet P. Z. (1998). Definition, diagnosis and classification of diabetes mellitus and its complications. Part 1: diagnosis and classification of diabetes mellitus provisional report of a WHO consultation. *Diabetic Medicine*.

[26] Chen C., Lu F. C., Department of Disease Control Ministry of Health, PR China (2004). The guidelines for prevention and control of overweight and obesity in Chinese adults. *Biomedical and Environmental Sciences*.

[27] Zhai Y., Zhao W. H., Chen C. M. (2010). Verification on the cut-offs of waist circumference for defining central obesity in Chinese elderly and tall adults. *Zhonghua Liu Xing Bing Xue Za Zhi*.

[28] Andersson T., Alfredsson L., Kallberg H., Zdravkovic S., Ahlbom A. (2005). Calculating measures of biological interaction. *European Journal of Epidemiology*.

[29] Knol M. J., Vander Weele T. J., Groenwold R. H., Klungel O. H., Rovers M. M., Grobbee D. E. (2011). Estimating measures of interaction on an additive scale for preventive exposures. *European Journal of Epidemiology*.

[30] American Diabetes Association (2012). Standards of medical care in diabetes—2012. *Diabetes Care*.

[31] Qin X., Li J., Zhang Y. (2012). Prevalence and associated factors of diabetes and impaired fasting glucose in Chinese hypertensive adults aged 45 to 75 years. *PLoS One*.

[32] Zhao Q., Zhen Q., Li Y. (2018). Prevalence and risk factors of impaired fasting glucose among adults in Northeast China: a cross-sectional study. *Endocrine Practice*.

[33] Li S., Guo S., He F. (2015). Prevalence of diabetes mellitus and impaired fasting glucose, associated with risk factors in rural Kazakh adults in Xinjiang, China. *International Journal of Environmental Research and Public Health*.

[34] Yu S., Sun Z., Zheng L., Guo X., Yang H., Sun Y. (2015). Prevalence of diabetes and impaired fasting glucose in hypertensive adults in rural China: far from leveling-off. *International Journal of Environmental Research and Public Health*.

[35] Wang B., Zhang M., Liu Y. (2018). Utility of three novel insulin resistance‐related lipid indices for predicting type 2 diabetes mellitus among people with normal fasting glucose in rural China. *Journal of Diabetes*.

[36] Lee J.-W., Lim N.-K., Park H.-Y. (2018). The product of fasting plasma glucose and triglycerides improves risk prediction of type 2 diabetes in middle-aged Koreans. *BMC Endocrine Disorders*.

[37] Ioachimescu A. G., Brennan D. M., Hoar B. M., Hoogwerf B. J. (2010). The Lipid Accumulation Product and All‐cause Mortality in Patients at High Cardiovascular Risk: A PreCIS Database Study. *Obesity*.

[38] Gu Z., Zhu P., Wang Q. (2018). Obesity and lipid-related parameters for predicting metabolic syndrome in Chinese elderly population. *Lipids in Health and Disease*.

[39] Nielsen S., Guo Z., Johnson C. M., Hensrud D. D., Jensen M. D. (2004). Splanchnic lipolysis in human obesity. *The Journal of Clinical Investigation*.

[40] Lemieux S., Prud'homme D., Bouchard C., Tremblay A., Després J. P. (1993). Sex differences in the relation of visceral adipose tissue accumulation to total body fatness. *The American Journal of Clinical Nutrition*.

[41] Kahn H. S., Cheng Y. J. (2008). Longitudinal changes in BMI and in an index estimating excess lipids among white and black adults in the United States. *International Journal of Obesity*.

[42] Song J., Chen X., Zhao Y., Mi J., Wu X., Gao H. (2018). Risk factors for prehypertension and their interactive effect: a cross- sectional survey in China. *BMC Cardiovascular Disorders*.

[43] Marcadenti A., Fuchs F. D., Moreira L. B., Gus M., Fuchs S. C. (2017). Adiposity phenotypes are associated with type-2 diabetes: LAP index, body adiposity index, and neck circumference. *Atherosclerosis*.

[44] Elffers T. W., de Mutsert R., Lamb H. J. (2017). Body fat distribution, in particular visceral fat, is associated with cardiometabolic risk factors in obese women. *PLoS One*.

[45] Higashiosaka I. (2014). Influence of age and gender on lipid accumulation product and its relation to diabetes mellitus in Japanese. *Clinica Chimica Acta*.

[46] Li R., Li Q., Cui M. (2017). Visceral adiposity index, lipid accumulation product and intracranial atherosclerotic stenosis in middle-aged and elderly Chinese. *Scientific Reports*.

[47] Lima-Martinez M. M., Blandenier C., Iacobellis G. (2013). Epicardial adipose tissue: more than a simple fat deposit?. *Endocrinología y Nutrición*.

[48] Preis S. R., Massaro J. M., Robins S. J. (2010). Abdominal subcutaneous and visceral adipose tissue and insulin resistance in the Framingham heart study. *Obesity*.

[49] Liu L., Feng J., Zhang G. (2018). Visceral adipose tissue is more strongly associated with insulin resistance than subcutaneous adipose tissue in Chinese subjects with pre-diabetes. *Current Medical Research and Opinion*.

[50] Rafiq R., Walschot F., Lips P. (2018). Associations of different body fat deposits with serum 25-hydroxyvitamin D concentrations. *Clinical Nutrition*.

[51] Madero M., Katz R., Murphy R. (2017). Comparison between different measures of body fat with kidney function decline and incident CKD. *Clinical Journal of the American Society of Nephrology*.

[52] Tang L., Zhang F., Tong N. (2016). The association of visceral adipose tissue and subcutaneous adipose tissue with metabolic risk factors in a large population of Chinese adults. *Clinical Endocrinology*.

[53] Nazare J. A., Smith J. D., Borel A. L. (2012). Ethnic influences on the relations between abdominal subcutaneous and visceral adiposity, liver fat, and cardiometabolic risk profile: the international study of prediction of Intra-Abdominal adiposity and its relationship with cardiometabolic Risk/Intra-Abdominal adiposity. *The American Journal of Clinical Nutrition*.

[54] Wolfgram P. M., Connor E. L., Rehm J. L. (2015). In nonobese girls, waist circumference as a predictor of insulin resistance is comparable to MRI fat measures and superior to BMI. *Hormone Research in Pædiatrics*.

[55] Perona J. S., Schmidt Rio-Valle J., Correa-Rodriguez M., Fernandez-Aparicio A., Gonzalez-Jimenez E. (2018). Waist circumference and abdominal volume index are the strongest anthropometric discriminators of metabolic syndrome in Spanish adolescents. *European Journal of Clinical Investigation*.

[56] Yusuf S., Hawken S., Ôunpuu S. (2005). Obesity and the risk of myocardial infarction in 27,000 participants from 52 countries: a case-control study. *The Lancet*.

[57] de Koning L., Merchant A. T., Pogue J., Anand S. S. (2007). Waist circumference and waist-to-hip ratio as predictors of cardiovascular events: meta-regression analysis of prospective studies. *European Heart Journal*.

[58] Cho J. H., Rhee E. J., Park S. E. (2019). The risk of myocardial infarction and ischemic stroke according to waist circumference in 21,749,261 Korean adults: a nationwide population-based study. *Diabetes and Metabolism Journal*.

[59] Boursier G., Sultan A., Molinari N. (2018). Triglycerides and glycated hemoglobin for screening insulin resistance in obese patients. *Clinical Biochemistry*.

[60] Cui J., Sun J., Wang W. (2017). The association of triglycerides and total cholesterol concentrations with newly diagnosed diabetes in adults in China. *Oncotarget*.

[61] Cui J., Sun J., Wang W. (2018). Triglycerides and total cholesterol concentrations in association with IFG/IGT in Chinese adults in Qingdao, China. *BMC Public Health*.

[62] Nguyen-Duy T. B., Nichaman M. Z., Church T. S., Blair S. N., Ross R. (2003). Visceral fat and liver fat are independent predictors of metabolic risk factors in men. *American Journal of Physiology-Endocrinology and Metabolism*.

[63] Ren Y., Liu Y., Sun X. (2017). Hypertriglyceridemia-waist and risk of developing type 2 diabetes: the rural Chinese cohort study. *Scientific Reports*.

[64] Zhao K., Yang S. S., Wang H. B., Chen K., Lu Z. H., Mu Y. M. (2018). Association between the hypertriglyceridemic waist phenotype and prediabetes in Chinese adults aged 40 years and older. *Journal Diabetes Research*.

[65] Roriz A. K., Passos L. C., de Oliveira C. C., Eickemberg M., Moreira Pde A., Sampaio L. R. (2014). Evaluation of the accuracy of anthropometric clinical indicators of visceral fat in adults and elderly. *PLoS One*.

[66] Ustulin M., Rhee S. Y., Chon S. (2018). Importance of family history of diabetes in computing a diabetes risk score in Korean prediabetic population. *Scientific Reports*.

[67] Wagner R., Thorand B., Osterhoff M. A. (2013). Family history of diabetes is associated with higher risk for prediabetes: a multicentre analysis from the German Center for Diabetes Research. *Diabetologia*.

[68] Sargeant L. A., Wareham N. J., Khaw K. T. (2000). Family history of diabetes identifies a group at increased risk for the metabolic consequences of obesity and physical inactivity in EPIC-Norfolk: a population-based study. *International Journal of Obesity and Related Metabolic Disorders*.

[69] Hilding A., Eriksson A. K., Agardh E. E. (2006). The impact of family history of diabetes and lifestyle factors on abnormal glucose regulation in middle-aged Swedish men and women. *Diabetologia*.

[70] Cullen M. W., Ebbert J. O., Vierkant R. A., Wang A. H., Cerhan J. R. (2009). No interaction of body mass index and smoking on diabetes mellitus risk in elderly women. *Preventive Medicine*.

[71] Bozorgmanesh M., Hadaegh F., Azizi F. (2010). Diabetes prediction, lipid accumulation product, and adiposity measures; 6-year follow-up: Tehran lipid and glucose study. *Lipids in Health and Disease*.

